# Potential molecular mechanisms for fruiting body formation of Cordyceps illustrated in the case of *Cordyceps sinensis*

**DOI:** 10.1080/21501203.2017.1365314

**Published:** 2017-08-30

**Authors:** Kun Feng, Lan-ying Wang, Dong-jiang Liao, Xin-peng Lu, De-jun Hu, Xiao Liang, Jing Zhao, Zi-yao Mo, Shao-ping Li

**Affiliations:** a State Key Laboratory of Quality Research in Chinese Medicine, University of Macau, Macao, China; b Department of Chemistry and Pharmacy, Zhuhai College of Jilin University, Zhuhai, China; c The State Key Laboratory of Respiratory Diseases, Guangzhou Medical University, Guangzhou, China; d Bino Beijing Limited, Beijing, China

**Keywords:** Proteomics, *Cordyceps sinensis*, fruiting body formation, molecular mechanism, signal pathway

## Abstract

The fruiting body formation mechanisms of *Cordyceps sinensis* are still unclear. To explore the mechanisms, proteins potentially related to the fruiting body formation, proteins from fruiting bodies, and mycelia of *Cordyceps* species were assessed by using two-dimensional fluorescence difference gel electrophoresis, and the differential expression proteins were identified by matrix-assisted laser desorption/ionisation tandem time of flight mass spectrometry. The results showed that 198 differential expression proteins (252 protein spots) were identified during the fruiting body formation of *Cordyceps* species, and 24 of them involved in fruiting body development in both *C. sinensis* and other microorganisms. Especially, enolase and malate dehydrogenase were first found to play an important role in fruiting body development in macro-fungus. The results implied that cAMP signal pathway involved in fruiting body development of *C. sinensis*, meanwhile glycometabolism, protein metabolism, energy metabolism, and cell reconstruction were more active during fruiting body development. It has become evident that fruiting body formation of *C. sinensis* is a highly complex differentiation process and requires precise integration of a number of fundamental biological processes. Although the fruiting body formation mechanisms for all these activities remain to be further elucidated, the possible mechanism provides insights into the culture of *C. sinensis*.

## Introduction

**Figure 1.**
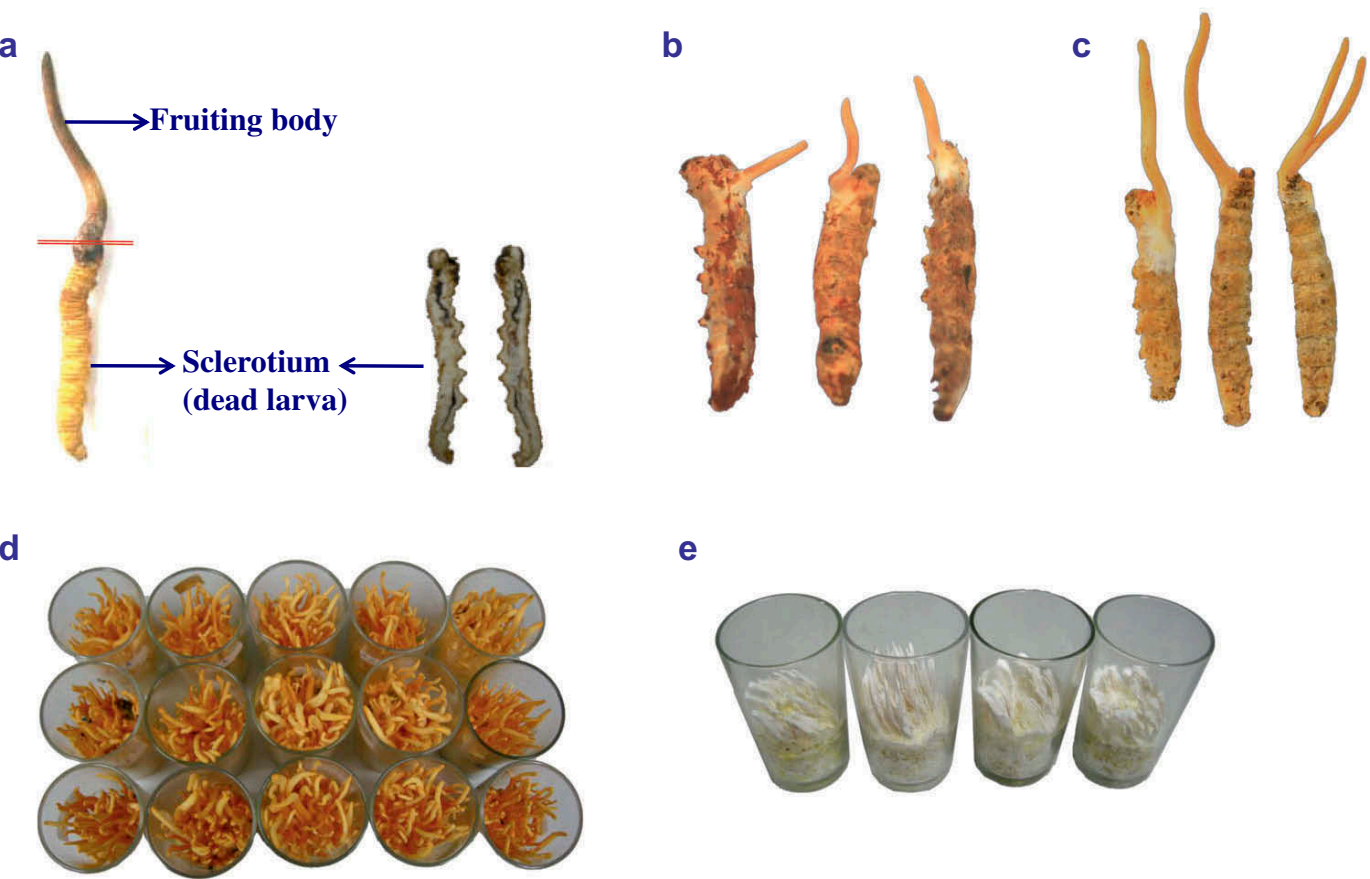
The pictures of (a) natural *Cordyceps sinensis*, (b) early and (c) late stages of *C. militaris*, cultured fruiting bodies of (d) *C. militaris* and (e) *C. memorabilis.*

*Cordyceps* is a large genus of entomogenous fungi with more than 400 species found world-wide, and the most famous and valuable species is *Cordyceps sinensis* (Berk.) Sacc. (Li et al. 2006a). Wild *C. sinensis* is also known as “Dong Chong Xia Cao” in Chinese or “Yartsa gunbu” in Tibetian, which means “Winter Worm Summer Grass” because of their appearance in different seasons (Paterson 2008) (Figure 1). *C. sinensis* has multiple beneficial effects on hepatic, renal, cardiovascular, immunologic, and nervous systems (Wang and Shiao 2000; Paterson 2008), and been used as highly prized herbal medicine and healthy food. Natural *C. sinensis* is found only in the soil of a prairie at an elevation of 3 000 to 5 000 m mainly in Tibet, Qinghai, Gansu, Sichuan, and Yunnan provinces in China. The worldwide demand for natural *C. sinensis* has been increasing continuously. With the reckless exploration, the annual harvest has been decreasing rapidly and resulting in serious habitat destruction (Li et al. 2011). The price of *C. sinensis* reached USD 13,000 per kg in 2008–2009 (Au et al. 2011), and the top quality *C. sinensis* rocketed up to USD 32,000 per kg in Hong Kong and San Francisco in late 2006 (Winkler 2008). Therefore, the cultured *C. sinensis* becomes an urgent need and inevitable trend. After several decades of efforts, 572 fungal strains of more than 37 genera have been isolated from natural *C. sinensis* (Zhang, Zhang et al. 2010, Zhang, Sun et al. 2010). Generally, the fungus of *Hirsutella sinensis* X.J. Liu, Y.L. Guo, Y.X. Yu & W. Zeng is recognised as the anamorph of *C. sinensis* (Chen et al. 2004; Li et al. 2006a; Zhong et al. 2010). Up to date, the molecular mechanisms, which are critical for cultivation of *C. sinensis*, of fruiting body development of *C. sinensis* are still unknown.

Proteins usually play important biological roles in regulating metabolic processes, signal transduction, small molecule or ion transportation, cell replication, and apoptosis (Gauci et al. 2011). The identification of differentially expressed proteins during fruiting body development could improve better understanding of *C. sinensis* formation. Proteomics is aimed at the large-scale and systematic characterisation of the entire protein complement of a cell line, tissue, or organism at a particular time, under a particular set of conditions (Graves and Haystead 2002; Beranova-Giorgianni 2003; Giepmans et al. 2006). Proteomics can be used as an important tool in helping to elucidate mechanisms of biological processes in a high-throughput mode. Classical two-dimensional electrophoresis (2DE) for protein isolation coupled with protein spot identification by mass spectrometry is the most widely adopted approach in proteomics studies (De Roos and McArdle 2008), but traditional 2DE is time-consuming, labour-intensive, limited sensitivity and prone to experimental errors, so this approach requires several replicate runs to overcome the gel-to-gel variations (Minden 2007; Chevalier 2010). In order to overcome the limitations of 2DE, a modified 2DE technique called fluorescence difference gel electrophoresis (DIGE) has been developed for direct quantitative measurements among differentially labelled samples using cyanine fluorescent dyes prior to gel electrophoresis and it is more accurate, sensitive, confident, reproducible and not limited by the distortion from gel-to-gel variation (Van Den Bergh and Arckens 2004; De Roos and McArdle 2008; Muroi et al. 2010).

To date, little has been known for the fruiting body formation mechanism and proteome of *C. sinensis* (Jin 2005; Kao 2006). The objective of this study is to unveil the fruiting body formation mechanism of *C. sinensis* as well as its related species based on differential protein expression of the fruiting body, sclerotium of *C. sinensis* and mycelium of *H. sinensis*, mature (late stage), immature (early stage) fruiting bodies and mycelium of *Paecilomyces militaris* (Kob.) Brown & Smith ex Liang, anamorph of *C. militaris* (L.: Fr.) Link (Liu et al. 2002), as well as fruiting body and mycelium of *Isaria farinose* (Holm ex S.F. Gray) Fr., anamorph of *C. memorabilis* (Ces.) Sacc. (Zimmermann 2008).

## Materials and methods

### Natural *C. sinensis*, fungal strains and materials

Natural fresh *C. sinensis*, including the fruiting body and sclerotium, were collected from Huzhu County, Qinghai Province of China. The fungal strain of *H. sinensis* was purchased by the Institute of Microbiology of Chinese Academy of Sciences, China. Fungal strain of *P. militaris* (anamorph of *C. militaris*) was gift from Zhangjiagang City Zanglian Biotechnology Co., Ltd., Jiangsu Province, China; and strain *Isaria farinosa* (anamorph of *C. memorabilis*) was isolated from infected caterpillar provided by Qinghai Academy of Animal and Veterinary Science, China, which was identified by the Institute of Microbiology of Chinese Academy of Sciences, China. Golden rabbit Thai fragrant rice purchased from San Miu Supermarket Limited in Macao, China; foxtail millet (*Setaria italica*) obtained from Yilan County Seed Company, Heilongjiang Province of China; silkworm larvae and silkworm pupa powder bought from Sericulture and Farm Produce Processing Research Institute, Guangzhou, China; mould liquid medium purchased from Guangdong Huankai Microbial Sci. & Tech. Co., Ltd., China.

### Fungal culture conditions and media

he fungi were cultured as the method described in our previous report (Feng et al. 2009) with modification. In brief, the fungal strains in tube slant were implanted into improved mould liquid medium (16.6 g mould liquid medium, the extract of 200.0 g fresh potato, and 2.0 g yeast extract in 1 l with Milli-Q water, pH 5.6). Conical flasks (500 ml) containing 150 ml of medium were inoculated with purified colony and incubated in an C24KC refrigerated incubator shaker (New Brunswick Scientific, USA) under 150 RPM at 16°C for *H. sinensis* or 22°C for *P. militaris* and *I. farinose* until plentiful mycelia balls presented. The mycelia were harvested by centrifugation, washed twice with sterile PBS buffer, and stored at 4ºC after lyophillisation. The fruiting body of *C. militaris, C. memorabilis* was cultured in improved rice medium (290 g Golden rabbit Thai fragrant rice, 290 g foxtail millet (*Setaria italica*), 50 g silkworm pupa powder, 16.6 g mould liquid medium and 1 l distilled water) sterilised at 121ºC for 30 min. Each cultivation bottle was inoculated with 8 ml of liquid seed and incubated in the dark at 22ºC for *C. militaris* and *C. memorabilis* with humidity levels of 70%, respectively. When the mycelia completely colonised the jar, the jar was exposed to fluorescent lamp (about 200 lx). After 3 to 5 days, the temperature was set a cycle of 22ºC for 12 h, and 12ºC for 12 h under a 12 h light/dark cycle condition to promote primordia formation. Once a large number of primordia were produced, the temperature was kept at 22ºC, and the relative humidity was kept at around 85% under a 12 h light/dark cycle condition for the formation of the fruiting body. The early and late stage fruiting bodies of *C. militaris* were cultured in fifth instar larvae of silkworm. In brief, the 5^th^ instar silkworm larva was surface sterilised with medical povidone-iodine swabs and then 0.3 ml of mycelial homogenate was injected under axenic conditions. The inoculated larvae were fed with fresh mulberry leaves at 22°C in the dark with 70% relative humidity. After the larva grew into stiff silkworm, the cultivation conditions were the same as the fruiting body of *C. militaris* grown on solid medium. When the fruiting body grew to approximate 2 cm (early stage) or produced spores (late stage), they were harvested and stored at −80°C.

### Extraction of proteins

The investigated materials (mycelia, worm or the fruiting body) were ground to a fine powder in liquid nitrogen using a mortar and pestle, added lysis buffer (containing 7 M urea, 2 M thiourea, 4% (w/v) CHAPS, 1% (w/v) DTT, 0.5% (v/v) IPG buffer pH 3–10, and 1 mM PMSF, from GE Healthacare) based on the modified Handbook 80–6429-60AC (GE Healthcare), and continued to grind to homogenate. The homogenate was transferred to a 1.5 ml Eppendorf tube and frozen in liquid nitrogen for 3 min, and then it was thawed in 37°C water for 3 min. For fully extracting the intracellular proteins, this step was repeated three times. After centrifugation at 28,113 × *g* for 30 min at 4°C, the supernatant was transferred to new tubes.

### Clean-up proteins

Proteins were purified with a 2-D Clean-Up Kit (GE Healthcare) according to the manufacturer’s instructions, revision 80–6486-60/Rev. CO/11–02. Briefly, proteins solution (200 μl) was mixed well with 600 μl of precipitant and incubated for 15 min on ice, then 600 μl co-precipitant was added and centrifuged at 28,113 × *g* for 5 min at 4°C. Added co-precipitant 4 times the size of the pellet after removing the supernatant, next centrifuged at 28,113 × *g* for 5 min. Pipetted enough Milli-Q water to disperse the pellet, and added 1 ml of pre-chilled wash buffer and 5 μl wash additive at −20°C for at least 30 min, vortexed for 20–30 s once every 10 min. The mixture was further centrifuged at 28,113 × *g* for 5 min at 4°C, and the supernatant was discarded and the pellet was allowed to dry briefly. The pellet was solubilised in lysis buffer without DTT and IPG buffer. Lastly, the protein solution was centrifuged at 28,113 × *g* for 20 min at 4ºC, and the supernatant was collected and stored at −80ºC. Prior to quantification, pH of protein samples was adjusted to 8.5 by using 1 M NaOH, as monitored by the pH Test Strip (4.5–10.0, Sigma). Finally, protein concentrations were determined with Bio-Rad Protein Assay Dye Reagent Concentrate (Bio-Rad) using BSA (2 mg ml^−1^) as the standard.

### Labelling of proteins with CyDye

All steps were operated in dark room. The proteome samples were labelled for DIGE analysis using Cy2, Cy3 and Cy5 CyDye™ DIGE Fluor minimal dye (GE Healthcare), respectively, according to the manufacturer manual (GE Healthcare). Cy2 was used to label an internal standard which was pooled equal amounts of each of all samples. Each 50 μg protein sample was labelled at a ratio of 400 pmol of dye on ice for 30 min, and the labelling reaction was terminated by adding 1 μl of 10 mM lysine and left on ice for 15 min. The three labelled samples were mixed into a single tube, and then both of extra 300 μg paired protein samples in a gel were added to the same tube, thus total of 750 μg protein samples were mixed in the tube and later could be used as preparative gel for spots picking. Equal volumes of 2× sample rehydration buffer (7 M urea, 2 M thiourea, 2% DTT, 4% CHAPS, 1% pH 3–10 NL IPG buffer (GE Healthacare), and 0.004% bromphenol blue) was added to the protein samples. Rehydration buffer (7 M urea, 2 M thiourea, 4% CHAPS, 1% DTT, 0.5% IPG buffer, and 0.004% bromphenol blue from Bio-Rad) was added to reach volumes to 450 μl for rehydration.

### Two-dimensional electrophoresis

The mixture was transferred to IPGbox (GE Healthcare), and ReadyStrip IPG Strips (24 cm, pH 5–8 from Bio-Rad) was put on the mixture with the gel side down. The gel was covered with DryStrip Cover Fluid (GE Healthcare), and rehydrated for 18 h at 20°C. After rehydration, the IPG strip was transferred to Ettan IPGphor Manifold of Ettan IPGphor 3 Isoelectric Focusing Unit (GE Healthcare). The first dimension isoelectric focusing (IEF) separation of 2DE was performed at 20°C with following sequential steps: 50 V rapid for 4 h; 150 V gradient for 2 h; 250 V gradient for 2 h; 500 V gradient for 2 h; 1,000 V gradient for 3.5 h; 5,000 V rapid for 1.5 h; 8,000 V rapid for 2 h; 10,000 V rapid for 70,000 Vh. After IEF, the strips were equilibrated in 50 mM Tris-HCl (pH 8.8), 6 M urea, 30% glycerol, 2% SDS, and 0.01% bromophenol blue with the addition of 2% DTT for 15 min in the dark. Subsequently, the strips were equilibrated with the same buffer with 2.5% (w/v) iodoacetamide instead of DTT for 15 min in the dark. Prior to preparation of SDS-polyacylamide gels, the longer low fluorescent glass plate was painted with PlusOne™ Repel-Silane ES (GE Healthcare) to assure gel release; while the shorter glass plate was painted with 4 ml PlusOne™ Bind-Silane (GE Healthcare) in 1 ml of acidic ethanol (0.5% acetic acid in 95% ethanol) to covalently attach the polyacrylamide gel to glass surface. SDS-PAGE was performed as second dimensional separation in 12.5% acrylamide gels in an Ettan™ DALT Six-Large Vertical System (GE Healthcare). The electrophoresis was performed at 15 mA per gel for 40 min, then 30 mA per gel until the bromophenol blue line reached the bottom of the gel at 10°C in the dark.

### Imaging and analysis

Cy2-, Cy3- and Cy5-labelled samples were acquired in an Ettan DIGE Imager (GE Healthcare) according to the manufacturer’s instructions. The images were checked for intensity during the acquisition process using ImageQuant™ TL software (GE Healthcare), and analysed by using DeCyder™ 2-D Differential Analysis Software v7.0 (GE Healthcare). After analysis, the gels were stained in 0.1% Coomassie Brilliant Blue-R250 solution in 50% ethanol and 10% acetic acid for 2.5 h, and destained in 25% ethanol and 8% acetic acid for 1.5 h. Then, they were washed with Milli-Q water and scanned on a UMAX PowerLook 2100XL scanner (UMAX Technologies). Protein spots at least 2-fold differences in expression level were automatically and accurately excised into 96-cell plates using Ettan Spot Picker (GE Healthcare). All parameters were set according to the manufacturer’s protocol.

### In-gel digestion and protein identification by MALDI TOF/TOF MS

Each excised protein slice was washed with 100 μl Milli-Q water (twice, for 2 × 5 min) on ice, and destained with 80 μl of 50 mM ammonium bicarbonate (Sigma) /acetonitrile (ACN, Sigma) (1:1) for 30 min at 37°C. The gel piece was incubated in 70 ul of acetonitrile until it was white and sticky. After removing solution, the gel piece was rehydrated in 2 μl of trypsin solution (2 μg Promega’s Sequencing Grade Modified Trypsin mixed with 140 μl of 25 mM ammonium bicarbonate containing 10% acetonitrile) on ice for 20 min. Excess trypsin was removed, the gel piece was covered with 20 μl of 25 mM ammonium bicarbonate containing 10% acetonitrile, and trypsinised at 37°C for 16 h. After digestion, the peptides were transferred into a new PCR tube, and the pellet was extracted with 30 μl of 0.1% trifluoroacetic acid (TFA, Sigma-Aldrich) in 67% acetonitrile at 37°C for 30 min, subsequently was ultrasonicated for 20 min at room temperature. This step was repeated again. Total extracts and the first digested peptides were mixed, vacuum-dried, and dissolved in 2 μl of 0.1% TFA in 30% acetonitrile. After in-gel digestion, 0.4 μl of the supernatant was spotted onto a MALDI plate (Opti-TOF^TM^ 384-well Insert, Applied Biosystems), and 0.4 μl of matrix solution (saturated solution of α-cyano-4-hydroxycinammic acid in 50% acetonitrile and 0.1% TFA) were added to the peptide and allowed to air-dry at room temperature. Tryptic peptides of 0.5 μl were analysed using a 4800 *plus* MALDI TOF/TOF Analyser (Applied Biosystems) with positive ion reflection mode, and standards (ABI 4700 Calibration Mixture, Applied Biosystems) were conducted to calibrate the spectrum to a mass tolerance within 0.1 Da. The parameters for database searching were peptide tolerance of 80–150 ppm; MS/MS tolerance of 0.2–0.3 Da; one missed cleavage; variable modifications of carbamidomethyl (Cys), oxidised (Met). GPS Explorer™ software v3.6 (Applied Biosystems) was used to search files in the National Centre for Biotechnology non-redundant (NCBInr) all species database, fungus database and insect database. All the identified proteins have MASCOT report total protein score C.I.% or total ion C.I.% greater than 95% and identification probability score at *p *< 0.05.

## Results and discussion

### Proteins in fruiting bodies, sclerotia and mycelia of *Cordyceps* species

#### Differential proteins in the fruiting body/sclerotia and fruiting body/mycelia from natural *C. sinensis*

**Figure 2.**
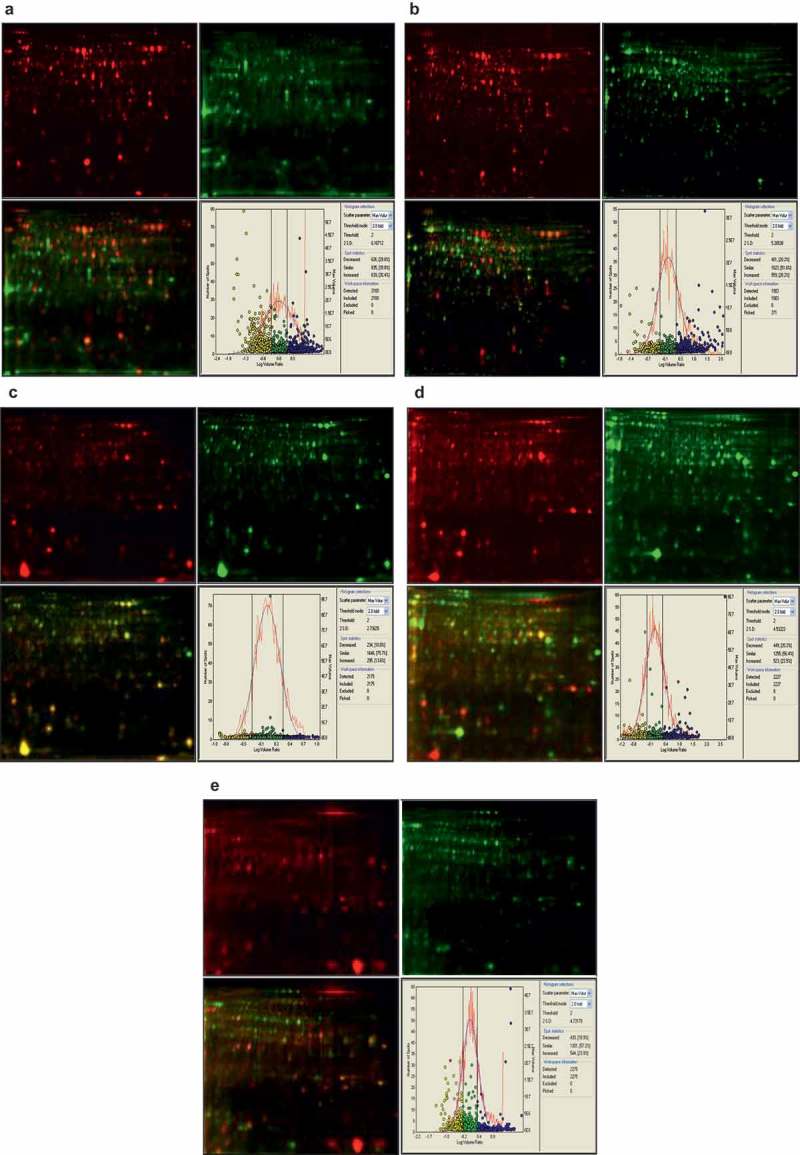
2 D-DIGE images of proteins from *Cordyceps* materials labeled with Cy3 (green in online versiton) or Cy5 (red in online versiton) and their merge (color in online versiton) and statistical analysis of differentially expressed spots. A. Fruiting body (red in online versiton) versus sclerotia (dead larvae, green in online versiton) of *C. sinensis*. B. Fruiting body (red in online versiton) of *C. sinensis* versusmycelia (green in online versiton) of *H. sinensis*. C. Later (red in online versiton) versus early (green in online versiton) stage fruiting body of *C. militaris*. D. Fruiting body (red in online versiton) versusmycelia (green in online versiton) of *P. militaris*. E. Fruiting body (red in online versiton) versus mycelia (green in online versiton) of *I. farinosa*. For gel image, pH, 5 to 8 linear from left to right; mass, ∼100 kDa to ∼10 kDa from top to bottom.

**Figure 3.**
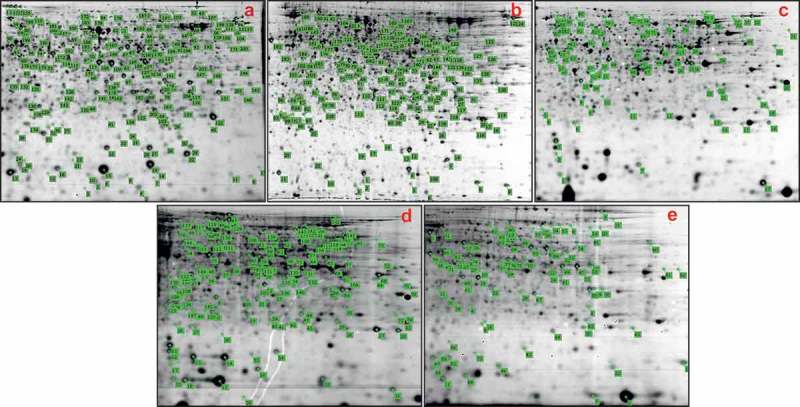
Picked high differential expression protein spots in gels. (a–e) the same as in Figure 2. For gel image, pH, 5 to 8 linear from left to right; mass, ~100 kDa to ~10 kDa from top to bottom. Green number indicates the protein spot ID.

Natural worm without infection of fungus of *Cordyceps* is difficult to obtain for the proteomics investigation due to specific life cycle of *C. sinensis* and the habitat. Therefore, the fruiting body and sclerotia (dead larvae) from natural *C. sinensis* were used, and 2100 spots were detected in both the fruiting body and sclerotium (Figure 2). The number was much more than 18 spots in natural *C. sinensis* (Jin 2005). Among the detected spots, 639 (30.4%) and 626 (29.8%) spots were up-regulated and down-regulated, respectively, in fruiting bodies of natural *C. sinensis* (Threshold mode: 2.0-fold). Among the detected proteins, only 62 proteins in 70 spots (36.8%) out of 190 picked spots (Figure 3) were successfully identified. The most likely reason for low ratio identification attributed to the limited genome or proteome database of *C. sinensis*, which is the greatest challenge for the study on proteome of *C. sinensis*. Indeed, no spot was identified except one protein, which was found in the fruiting body of natural *C. sinensis* rather than mycelia of *Hirsutella sinensis*, had high similarity to protein of hypothetical protein AN8043.2, putative fimbrial usher or UDP-N-acetylglucosamine pyrophosphorylase according to 10 amino acids sequence of its N-terminal (Jin 2005). Our results confirmed that UDP-N-acetylglucosamine pyrophosphorylase compared to sclerotium was up-regulated in the fruiting body of *C. sinensis*. Besides, only serine protease among the identified proteins was reported in an entomopathogenic fungus CS2 from *C. sinensis* (Zhang et al. 2008). The other proteins were firstly identified in *C. sinensis*. For the identified proteins, 6 proteins, such as serine protease, GAF domain protein, predicted similar to Actin-5C isoform 1, actin 6, beta actins, heat shock 70 kd protein cognate 1, were down-regulated, while 53 proteins, including acetaldehyde dehydrogenase, enolase, tubulins, eukaryotic initiation factor 4A, elongation factor 2, elongation factor 3, cobalamin-independent methionine synthase, fructose-bisphosphate aldolase, inorganic pyrophosphatase, UTP-glucose-1-phosphate uridylyltransferase, vacuolar ATP synthase catalytic subunit A, malate dehydrogenase, *O*-acetylhomoserine sulfhydrylase, mannose-1-phosphate guanyltransferase, septin, rab GDP-dissociation inhibitor, T-complex protein 1 subunit zeta, heat shock 70 kDa protein, putative Hsp70 chaperones, etc. were up-regulated in the fruiting body of *C. sinensis* (Table 1).

**Table 1.** Differentially expressed proteins during fruiting body formation of *Cordyceps* were identified by MALDI-TOF/TOF MS.

			Protein	Volume	Accession	*Mr*		Protein	Protein Score	Total Ion	Total Ion
No.	Protein Name	Group	ID	Ratio	No.	kDa	P*I*	Score	C. I. %	Score	C. I. %
1	Heat shock protein 90 [*Humicola fuscoatra*]	A/A/C/C/C/D	106/190/64/65/83/50	−2.05/-2.53/5.89/2.30/4.19/-2.04	gi|194,716,766	79.5	4.90	219/146/101/93/99/90	100/100/100/99.995/99.999/99.991	94/51/59/33/79/50	99.999/74.776/99.963/82.128/100/99.656
2	Hypothetical protein FG00857.1 [*Gibberella zeae* PH-1]	A/A/A/B	152/154/155/75	5.23/4.94/3.29/-4.29	gi|46,107,948	60.3	4.94	73/72/120/144	60.686/55.888/100/100	65/65/107/115	99.131/99.157/100/100
3	Cobalamin-independent methionine synthase [*Epichloe festucae*]	A/A/C/C	94/158/36/72	9.51/6.40/-2.49/2.38	gi|34,500,101	77.3	6.31	129/113/173/86	100/99.996/100/98.284	25/-/115/26	0/-/100/0
4	HS70_NEUCR Heat shock 70 kDa protein (HSP70) [*Gibberella zeae* PH-1]	A/A/C	64/77/85	15.52/12.79/-2.49	gi|46,107,910	71.1	5.00	367/334/375	100/100/100	258/256/271	100/100/100
5	Hypothetical protein FG09893.1 [*Gibberella zeae* PH-1]	A/B/B	67/154/160	14.68/4.21/9.63	gi|46,136,755	52.4	5.25	164/150/165	100/100/100	106/102/105	100/100/100
6	Malate dehydrogenase, mitochondrial precursor [*Neurospora crassa* OR74A]	A/B/C	145/51/20	7.19/20.14/-2.78	gi|85,109,459	34.3	5.56	138/182/262	100/100/100	90/124/187	99.996/100/100
7	UTP-glucose-1-phosphate uridylyltransferase [*Neurospora crassa* OR74A]	A/A	93/192	9.88/2.37	gi|164,427,705	58.2	6.59	167/157	100/100	89/63	99.996/98.107
8	Hsp70 chaperone (HscA), putative [*Talaromyces stipitatus* ATCC 10,500]	A/A	95/102	9.25/8.07	gi|242,798,753	52.7	5.84	253/194	100/100	217/152	100/100
9	Beta actin [*Mamestra brassicae*]	A/A	175/176	−6.03/-7.60	gi|157,927,723	41.8	5.23	431/372	100/100	253/195	100/100
10	Enolase BAC82549-*Penicillium chrysogenum* [*Penicillium chrysogenum* Wisconsin 54–1255]	A/B	91/177	10.21/5.23	gi|255,938,796	47.2	5.26	157/185	100/100	95/131	100/100
11	MPG1_TRIRE RecName: Full = Mannose-1-phosphate guanyltransferase	A/B	203/132	5.46/-2.65	gi|74,582,503	40.3	6.23	90/160	99.216/100	18/105	0/100
12	Heat shock 70 kd protein cognate 1 [*Magnaporthe oryzae* 70–15]	A/C	184/61	−3.18/3.93	gi|145,605,667	57.0	4.95	115/151	100/100	58/47	99.957/99.349
13	Rab GDP-dissociation inhibitor [*Neurospora crassa* OR74A]	A/C	54/67	19.29/-2.56	gi|85,105,909	51.4	5.33	97/137	99.861/100	45/117	58.762/100
14	Hypothetical protein FG06932.1 [*Gibberella zeae* PH-1]	A/E	53/71	19.56/-2.19	gi|46,125,109	46.9	6.52	82/88	95.689/98.637	61/49	96.720/44.658
15	Tubulin beta chain; AltName:Full = Beta-tubulin	A	45	44.92	gi|135,480	50.0	4.76	367	100	99	100
16	Hypothetical protein C34G6.1 [*Caenorhabditis elegans*]	A	58	17.48	gi|25,144,188	196.6	6.00	83	96.331		
17	Heat shock protein 90 [*Metarhizium anisopliae*]	A	59	17.38	gi|88,766,397	80.1	4.98	149	100	35	0
18	Eukaryotic initiation factor 4A [*Sclerotinia sclerotiorum* 1980]	A	60	17.09	gi|156,057,455	44.9	5.14	150	100	64	99.060
19	Hypothetical protein [*Podospora anserina* S mat+]	A	63	16.56	gi|171,690,144	54.0	5.77	57	83.850	48	99.564
20	Acetaldehyde dehydrogenase [*Ophiocordyceps heteropoda*]	A	72	13.80	gi|118,596,530	32.0	7.75	147	100	86	99.995
21	Beta-tubulin [*Chaetosphaerella phaeostroma*]	A	74	13.61	gi|59,894,499	36.3	5.62	171	100		
22	UDP-N-acetylglucosamine pyrophosphorylase [*Neurospora crassa* OR74A]	A	75	13.25	gi|85,111,786	53.6	5.19	124	100	87	99.996
23	Vacuolar ATP synthase catalytic subunit A [*Neurospora crassa* OR74A]	A	76	12.97	gi|85,103,674	67.1	5.32	187	100	108	100
24	Elongation factor 3 [*Neurospora crassa* OR74A]	A	82	12.28	gi|85,107,753	117.0	5.83	82	95.689	18	0
25	Fructose-bisphosphate aldolase [*Coccidioides posadasii*]	A	87	11.24	gi|9,837,587	13.4	6.82	97	99.847	74	99.880
26	Conserved hypothetical protein [*Magnaporthe oryzae* 70–15]	A	89	10.58	gi|145,606,158	59.8	5.23	72	99.465	17	0
27	Hsp70 chaperone BiP/Kar2, putative [*Talaromyces stipitatus* ATCC 10,500]	A	90	10.33	gi|242,764,265	73.5	4.92	137	100	97	100
28	Unnamed protein product [*Podospora anserina*]	A	97	8.99	gi|171,689,612	45.6	5.25	94	99.673	30	0
29	Insect origin recognition complex subunit, putative [*Pediculus humanus corporis*]	A	98	8.96	gi|212,506,989	64.5	5.61	30	0	30	98.597
30	Conserved hypothetical protein [*Chaetomium globosum* CBS 148.51]	A	107	7.42	gi|116,200,814	56.8	5.78	190	100	80	99.969
31	Hypothetical protein MGG_00341 [*Magnaporthe oryzae* 70–15]	A	181	7.16	gi|39,975,025	63.2	5.18	52	44.002	41	97.943
32	Ribosomal L18ae protein family [*Aspergillus clavatus* NRRL 1]	A	114	6.80	gi|119,397,850	23.7	10.33	68	98.746		
33	Inorganic pyrophosphatase [*Neurospora crassa* OR74A]	A	126	6.68	gi|164,428,710	32.6	5.28	145	100	107	100
34	*O*-acetylhomoserine sulfhydrylase [*Neotyphodium coenophialum*]	A	170	6.46	gi|121,551,073	42.6	6.28	208	100	117	100
35	PSA2_NEUCR Probable proteasome subunit alpha type 2 [*Gibberella zeae* PH-1]	A	149	6.46	gi|46,117,136	30.5	4.99	105	99.976	29	0
36	Hypothetical protein FG05150.1 [*Gibberella zeae* PH-1]	A	161	6.35	gi|46,121,543	49.6	5.82	104	99.969	33	0
37	Septin [*Exophiala dermatitidis*]	A	147	6.30	gi|91,719,120	38.4	8.19	88	98.757	50	78.232
38	T-complex protein 1 subunit zeta [*Neurospora crassa* OR74A]	A	167	6.02	gi|85,091,533	58.8	5.83	106	99.981	15	0
39	Hypothetical protein Igni_0048 [*Ignicoccus hospitalis* KIN4/I]	A	151	5.54	gi|156,936,843	15.2	8.36	82	95.163		
40	Hypothetical protein FG06803.1 [*Gibberella zeae* PH-1]	A	39	5.10	gi|46,124,851	26.5	7.71	93	99.598	33	0
41	Actin [*Neurospora crassa OR74A*]	A	115	5.05	gi|164,426,508	41.6	5.45	394	100	238	100
42	Hypothetical protein MGG_00135 [*Magnaporthe grisea* 70–15]	A	186	4.57	gi|39,975,437	59.2	5.10	110	99.992	48	52.875
43	Hypothetical protein CIMG_09361 [*Coccidioides immitisimmitis* RS]	A	164	4.55	gi|119,174,825	26.8	5.37	143	100	110	100
44	Hypothetical protein MGG_07060 [*Magnaporthe oryzae* 70–15]	A	199	4.54	gi|39,971,489	60.0	4.97	114	100	55	99.915
45	Unnamed protein product [*Mus musculus*]	A	141	4.51	gi|12,842,861	22.6	10.04	82	95.273		
46	Elongation factor 2 [Culex quinquefasciatus]	A	148	4.49	gi|170,070,172	20.8	9.51	82	95.273		
47	YALI0F15587p [*Yarrowia lipolytica*]	A	119	4.34	gi|50,556,104	35.7	5.50	157	100	129	100
48	Hypothetical protein [*Podospora anserina* S mat+]	A	197	4.22	gi|171,681,451	75.4	8.96	72	99.512		
49	70 kDa heat shock protein [*Paracoccidioides brasiliensis*]	A	180	4.20	gi|31,324,921	73.7	5.92	137	100	82	99.985
50	Hypothetical protein [*Podospora anserina* S mat+]	A	144	4.01	gi|171,683,445	41.3	5.91	64	96.384	35	88.743
51	Chorismate binding enzyme [*Burkholderia thailandensis* MSMB43]	A	196	3.98	gi|167,837,750	69.4	5.97	84	96.948		
52	Conserved hypothetical protein [*Magnaporthe grisea*70–15]	A	168	3.61	gi|145,616,104	54.1	6.94	148	100	96	100
53	Hypothetical protein [*Podospora anserina* S mat+]	A	194	3.57	gi|171,688,652	64.1	8.67	75	99.719	39	96.641
54	Hypothetical protein FG05222.1 [*Gibberella zeae* PH-1]	A	143	3.26	gi|46,121,687	27.9	5.81	148	100	129	100
55	Molybdopterin biosynthesis protein [*Sulfitobacter* sp. NAS-14.1]	A	187	2.18	gi|83,954,363	33.6	5.64	69	0	63	98.767
56	Proteasome component PUP3 [*Neurospora crassa* OR74A]	A	36	2.07	gi|164,427,141	21.2	5.11	161	100	108	100
57	GAF domain protein [*Campylobacterales bacterium* GD 1]	A	182	−3.72	gi|254,458,884	73.4	5.46	92	99.549		
58	Predicted: similar to Actin-5C isoform 1 [*Apis mellifera*]	A	129	−7.07	gi|48,137,684	41.8	5.30	244	100	145	100
59	Beta actin [*Pseudopleuronectes americanus*]	A	18	−13.09	gi|3,452,279	13.4	5.46	287	100	136	100
60	Actin 6 [*Aedes aegypti*]	A	173	−17.77	gi|71,383,976	41.8	5.23	481	100	336	100
61	Actin [*Amblyomma americanum*]	A	174	−24.85	gi|196,476,734	21.1	5.27	278	100	148	100
62	Serine protease [*Ophiocordyceps sinensis*]	A	121	−28.96	gi|161,897,707	40.3	6.66	171	100	138	100
63	Hypothetical protein [*Podospora anserina* S mat+]	B/B/B/C/D	190/191/192/81/97	−3.27/-4.22/-4.73/-2.64-/-3.87/	gi|171,690,628	72.9	5.88	182/190/144/153/135	100/100/100/100/100	84/120/70/67/35	100/100/99.996/99.995/89.628
64	GTP-binding nuclear protein Ran, putative [*Aspergillus clavatus* NRRL 1]	B/B	28/139	3.26/3.31	gi|119,396,524	23.6	6.44	209/140	100/100	103/52	100/99.831
65	Hypothetical protein FG05454.1 [*Gibberella zeae* PH-1]	B/B	128/142	−4.16/-2.37	gi|46,122,153	45.3	6.78	147/107	100/99.985	53/22	89.230/0
66	Hypothetical protein [*Podospora anserina* S mat+]	B/C	184/62	−4.58/-3.82	gi|171,683,195	43.7	5.13	237/208	100/100	91/118	100/100
67	Acetaldehyde dehydrogenase [*Cordyceps militaris*]	B/E	153/93	6.78/-3.68	gi|118,596,538	31.9	8.22	108/253	99.988/100	49/105	72.432/100
68	FDH_NEUCR RecName: Full = Formate dehydrogenase	B/E	144/11	−4.94/14.53	gi|729,469	40.9	5.93	61/62	93.268/94.774	42/54	98.200/99.853
69	Hypothetical protein [*Podospora anserina* S mat+]	B	34	18.28	gi|171,691,500	18.1	4.39	63	95.752	48	99.186
70	Mago nashi protein [*Neurospora crassa* OR74A]	B	108	14.37	gi|85,085,322	18.1	6.10	90	99.159	25	0
71	Mannitol-1-phosphate 5-dehydrogenase [*Bacillus clausii* KSM-K16]	B	61	13.91	gi|56,964,690	41.3	5.22	98	99.887	78	99.968
72	Hypothetical protein MGG_13315 [*Magnaporthe oryzae* 70–15]	B	114	12.85	gi|145,603,837	25.7	9.51	63	95.752		
73	Hypothetical protein FG05282.1 [*Gibberella zeae* PH-1]	B	22	8.29	gi|46,121,809	22.2	5.28	128	100	77	99.929
74	Heat shock 70 kDa protein [*Chaetomium globosum* CBS 148.51]	B	88	6.61	gi|116,200,213	71.4	5.01	255	100	144	100
75	Elongation factor 2; Short = EF-2	B	91	6.41	gi|189,045,117	93.2	6.24	130	100	86	100
76	Molecular chaperone Hsp70 [*Aspergillus clavatus* NRRL 1]	B	89	6.34	gi|119,397,564	69.6	5.07	98	99.999	28	39.945
77	Serine protease [*Scytalidium thermophilum*]	B	29	5.73	gi|300,250,850	24.8	7.86	52	46.522	42	96.705
78	Immunoglobulin heavy chain-binding protein homolog [*Gibberella zeae* PH-1]	B	25	4.87	gi|46,135,911	74.5	5.08	87	98.398	33	0
79	Hypothetical protein [*Podospora anserina* S mat+]	B	133	4.46	gi|171,684,365	38.2	6.67	58	85.606	47	99.423
80	Hypothetical protein [*Penicillium chrysogenum* Wisconsin 54–1255]	B	87	3.94	gi|255,950,542	39.8	9.59	52	39.997	43	99.479
81	Cell division control protein 3 [*Neurospora crassa* OR74A]	B	41	3.54	gi|164,423,542	52.1	7.21	123	100	63	99.103
82	Hypothetical protein BRAFLDRAFT_84624 [*Branchiostoma floridae*]	B	103	3.51	gi|219,449,381	423.9	5.83	82	95.051		
83	Hypothetical protein ATEG_02453 [*Aspergillus terreus* NIH2624]	B	136	2.89	gi|115,388,251	39.1	6.46	93	99.616	73	99.912
84	5ʹ-Methylthioadenosine phosphorylase (Meu1), putative [*Aspergillus clavatus* NRRL 1]	B	37	2.85	gi|119,396,242	33.9	5.95	55	70.612	50	99.496
85	Pyruvate kinase [*Heliobacterium modesticaldum* Ice1]	B	46	2.83	gi|167,628,213	63.2	5.60	83	96.496		
86	Hypothetical protein [*Podospora anserina* S mat+]	B	52	2.77	gi|171,681,866	38.9	6.01	74	99.670	59	99.951
87	Beta-tubulin [*Magnaporthe oryzae* 70–15]	B	181	2.67	gi|39,974,499	49.9	4.80	85	99.973	22	0
88	Hypothetical protein FG09282.1 [*Gibberella zeae* PH-1]	B	135	2.67	gi|46,134,285	36.6	6.18	93	99.641	39	0
89	Subtilisin-like serine protease PR1H [*Metarhizium anisopliae*]	B	32	2.54	gi|254,351,261	53.9	6.21	59	89.081	50	99.548
90	Hypothetical protein [*Podospora anserina* S mat+]	B	188	2.33	gi|171,692,279	64.2	5.22	161	100	39	95.428
91	Guanine nucleotide-binding protein beta subunit-like protein [*Chaetomium globosum* CBS 148.51]	B	118	2.30	gi|116,201,077	35.1	6.55	174	100	101	100
92	Actin [*Paecilomyces lilacinus*]	B	113	2.16	gi|283,854,632	41.6	5.45	277	100	102	100
93	Hypothetical protein FG08593.1 [*Gibberella zeae* PH-1]	B	36	2.07	gi|46,128,431	25.9	6.70	103	99.962	57	94.689
94	Actin	B	23	2.04	gi|239,938,589	41.6	5.63	206	100	148	100
95	Transaldolase [*Magnaporthe grisea* 70–15]	B	45	−2.05	gi|39,970,315	35.6	5.38	243	100	196	100
96	Conserved hypothetical protein [*Chaetomium globosum* CBS 148.51]	B	73	−2.06	gi|116,201,583	37.8	5.76	192	100	72	99.848
97	Conserved hypothetical protein [*Magnaporthe oryzae* 70–15]	B	47	−2.12	gi|39,973,499	34.2	5.62	160	100	100	100
98	Hypothetical protein FG00505.1 [*Gibberella zeae* PH-1]	B	95	−2.24	gi|46,107,244	21.8	4.84	185	100	165	100
99	UDP-glucose pyrophosphorylase [*Phoma herbarum*]	B	123	−2.32	gi|159,459,918	57.8	7.23	101	100	87	100
100	Pc20g01500 [*Penicillium chrysogenum* Wisconsin 54–1255]	B	58	−2.51	gi|255,943,883	38.4	5.40	60	90.490	51	99.686
101	Hypothetical protein [*Entamoeba dispar* SAW760]	B	161	−2.64	gi|167,387,459	57.7	7.16	92	99.569	42	0
102	Ketol-acid reductoisomerase, mitochondrial precursor [*Neurospora crassa* OR74A]	B	49	−2.76	gi|85,102,477	44.6	8.52	142	100	97	100
103	Hypothetical protein [*Magnaporthe oryzae* 70–15]	B	180	−2.87	gi|39,968,579	16.9	9.24	46	0	41	98.288
104	Conserved hypothetical protein [*Magnaporthe oryzae* 70–15]	B	146	−2.96	gi|145,606,056	59.4	6.06	88	99.987	27	40.865
105	Putative RNA polymerase Rpb1, domain 2 [uncultured marine crenarchaeote HF4000_ANIW93I24]	B	59	−3.19	gi|167,042,230	140.3	7.81	84	97.217		
106	Pc18g01770 [*Penicillium chrysogenum* Wisconsin 54–1255]	B	97	−3.45	gi|255,942,505	26.6	5.80	72	99.440	56	99.945
107	Chorismate mutase [*Pyrenophora tritici-repentis* Pt-1C-BFP]	B	80	−3.59	gi|189,206,279	30.5	5.63	113	99.996	68	99.667
108	ATPB_NEUCR ATP synthase beta chain, mitochondrial precursor [*Gibberella zeae* PH-1]	B	182	−3.71	gi|46,116,940	54.9	5.40	229	100	106	100
109	ATP-citrat-lyase [*Gibberella pulicaris*]	B	152	−3.71	gi|7,159,697	53.0	5.57	195	100	149	100
110	Putrescine aminopropyltransferase [*Saccharomyces cerevisiae* YJM789]	B	111	−3.80	gi|151,942,852	33.3	5.33	117	99.998	45	54.649
111	Conserved hypothetical protein [*Magnaporthe oryzae* 70–15]	B	173	−4.80	gi|145,612,487	46.6	5.33	136	100	67	99.997
112	Hypothetical protein MGG_07268 [*Magnaporthe oryzae* 70–15]	B	129	−5.04	gi|145,612,637	46.4	7.03	87	99.981	34	90.103
113	Pc20g08020 [*Penicillium chrysogenum* Wisconsin 54–1255]	B	55	−5.82	gi|255,945,115	226.4	7.77	48	0	39	95.150
114	Thioredoxin peroxidase [*Ostertagia ostertagi*]	B	93	−6.95	gi|18,152,531	21.4	5.95	96	99.807	36	0
115	Conserved hypothetical protein [*Magnaporthe oryzae* 70–15]	B	50	−7.20	gi|39,970,291	29.6	6.14	72	99.386	29	27.759
116	Hypothetical protein [*Podospora anserina* S mat+]	B	120	−7.66	gi|171,677,424	35.0	6.55	124	100	39	97.853
117	PX domain-containing protein [*Toxoplasma gondii* ME49]	B	109	−8.24	gi|237,832,101	267.2	7.71	85	97.631		
118	Hsp70 chaperone (HscA), putative [*Talaromyces stipitatus* ATCC 10,500]	B	164	−9.73	gi|242,798,748	64.7	5.33	299	100	268	100
119	Unnamed protein product [*Podospora anserina* S mat+]	B	127	−11.01	gi|170,940,277	52.7	8.50	117	100	56	99.919
120	Hypothetical protein MGG_13201 [*Magnaporthe oryzae* 70–15]	B	183	−15.99	gi|145,603,296	16.5	9.79	64	96.778		
121	Hypothetical protein [*Penicillium chrysogenum* Wisconsin 54–1255]	B	19	−18.07	gi|255,940,706	15.8	9.75	66	97.666		
122	Putative enolase [*Beauveria bassiana*]	C/C/D/D/E/E/E	51/53/55/58/27/56/58/	2.45/3.36/-2.21/6.30/6.13/3.44/3.39	gi|110,592,112	47.2	5.07	253/159/471/292/166/125/103	100/100/100/100/100/100/99.962	182/117/386/212/97/52/57	100/100/100/100/100/81.499/91.839
123	6-phosphogluconate dehydrogenase [*Aspergillus clavatus* NRRL 1]	C/D/D/D	29/64/75/87	3.62/-4.11/6.78/-6.06	gi|119,396,136	56.0	6.05	89/153/79/70	99.990/100/99.883/99.209	60/109/51/51	99.958/100/99.753/99.753
124	Acetaldehyde dehydrogenase [*Cordyceps militaris*]	C/C/C	28/42/26	−2.62/2.05/3.95	gi|118,596,536	31.9	8.22	410/264/202	100/100/100	240/112/74	100/100/99.917
125	Guanine nucleotide-binding protein subunit beta-like protein [*Neurospora crassa*]	C/C	18/22	2.69/-2.69	gi|3,023,852	35.1	6.79	145/126	100/100	104/70	100/99.998
126	Actin [*Gaeumannomyces graminis*]	C/C	11/49	2.75/-2.81	gi|37,722,096	41.6	5.45	311/493	100/100	202/338	100/100
127	Hsp70 chaperone (HscA), putative [*Aspergillus clavatus* NRRL 1]	C/D	80/98	−3.32/-3.17	gi|119,404,708	66.9	5.19	306/182	100/100	285/156	100/100
128	Hypothetical protein MGG_06270 [*Magnaporthe oryzae* 70–15]	C/D	52/5	−2.86/-4.00	gi|39,976,735	38.3	5.18	100/63	100/96.036	86/54	100/99.906
129	Spermidine synthase [*Lodderomyces elongisporus* NRRL YB-4239]	C	8	6.51	gi|149,239,971	33.9	5.18	114	99.997	82	99.980
130	Heat shock protein 70 [*Paracoccidioides brasiliensis*]	C	4	5.25	gi|14,538,021	70.8	5.05	259	100	237	100
131	Heat shock protein 82 [*Aspergillus terreus* NIH2624]	C	89	4.09	gi|115,432,960	79.8	4.97	138	100	23	0
132	Pc22g21330 [*Penicillium chrysogenum* Wisconsin 54–1255]	C	6	3.99	gi|255,950,526	93.8	8.65	63	96.036		
133	Conserved hypothetical protein [*Magnaporthe oryzae* 70–15]	C	27	3.82	gi|39,953,501	34.3	6.85	188	100	104	100
134	Ribonuclease R [*Shewanella frigidimarina* NCIMB 400]	C	54	3.71	gi|114,564,516	92.8	8.70	84	97.018		
135	ATP synthase beta chain, mitochondrial precursor [*Chaetomium globosum* CBS 148.51]	C	58	3.53	gi|116,204,743	55.6	5.10	216	100	135	100
136	Hypothetical protein FG05315.1 [*Gibberella zeae* PH-1]	C	21	3.30	gi|46,121,875	44.8	6.19	100	99.923	35	0
137	Heat shock 70 kDa protein [*Ajellomyces capsulatus* NAm1]	C	45	3.22	gi|154,285,930	66.9	5.44	155	100	127	100
138	Hypothetical protein [*Podospora anserina* S mat+]	C	17	2.56	gi|171,694,267	95.9	5.22	73	99.585		
139	Inorganic diphosphatase, putative [*Aspergillus flavus* NRRL3357]	C	10	2.39	gi|238,484,693	43.6	7.06	168	100	89	99.997
140	Septin [*Aspergillus clavatus* NRRL 1]	C	63	2.29	gi|119,402,350	43.1	5.03	135	100	75	100
141	Predicted protein [*Physcomitrella patens* subsp. patens]	C	50	2.00	gi|168,041,049	10.9	5.57	84	96.804		
142	Zinc finger homeodomain 4 (predicted) [*Rattus norvegicus*]	C	73	−2.09	gi|149,048,501	222.6	5.85	82	95.486		
143	Beta-tubulin [*Botryotinia fuckeliana*]	C	60	−2.12	gi|1,002,511	49.7	4.88	296	100	108	100
144	Predicted protein [*Postia placenta* Mad-698-R]	C	82	−2.20	gi|242,222,974	56.5	10.73	63	95.234		
145	Heat shock protein 60 [*Gibberella zeae* PH-1]	C	95	−2.22	gi|46,123,737	61.4	5.57	288	100	216	100
146	Predicted protein [*Nematostella vectensis*]	C	66	−2.24	gi|156,372,872	462.0	6.09	87	98.573		
147	Poly(A) RNA binding protein [*Epichloe festucae*]	C	78	−2.41	gi|170,674,510	79.9	5.58	206	100	153	100
148	Hypothetical protein TRIADDRAFT_59511 [*Trichoplax adhaerens*]	C	77	−2.43	gi|196,011,279	709.9	5.91	98	99.867		
149	Predicted protein [*Nematostella vectensis*]	C	38	−2.47	gi|156,399,827	17.4	5.95	90	99.179		
150	Heat shock protein 70–2 [*Nicotiana tabacum*]	C	84	−2.62	gi|38,325,813	71.2	5.07	262	100	124	100
151	Beta-tubulin [*Fusarium sporotrichioides*]	C	59	−2.69	gi|269,978,742	37.3	5.53	135	100	33	86.749
152	Predicted protein [*Thalassiosira pseudonana* CCMP1335]	C	92	−4.37	gi|224,006,584	213.1	5.46	84	97.217		
153	GLYC_NEUCR Serine hydroxymethyltransferase [*Gibberella zeae* PH-1]	C	30	−4.41	gi|46,123,825	54.3	6.74	121	100		
154	Chitin deacetylase, putative [*Aspergillus clavatus* NRRL 1]	D/D	6/121	11.32/11.41	gi|119,396,283	53.4	6.30	49/60	0/90.707	42/53	97.245/99.850
155	Hypothetical protein MGG_00707 [*Magnaporthe oryzae* 70–15]	D/D	60/62	8.38/6.28	gi|39,974,293	44.9	5.89	80/62	99.907/95.123		
156	Hypothetical protein [*Podospora anserina* S mat+]	D/D	79/80	−2.56/-7.18	gi|171,695,892	52.3	5.36	84/70	99.965/99.172		
157	Glyceraldehyde-3-phosphate dehydrogenase [*Trichoderma koningii*]	D/D	67/68	−2.88/-2.25	gi|422,228	36.0	6.28	100/86	100/99.979	59/56	97.308/99.948
158	Hypothetical protein MGG_13200 [*Magnaporthe oryzae* 70–15]	D	150	3.00	gi|145,603,294	35.6	5.45	88	99.988		
159	Inosine-adenosine-guanosine-nucleoside hydrolase [*Trypanosoma brucei brucei*]	D	66	2.88	gi|2,645,495	35.8	5.23	82	95.051		
160	Glutathione synthetase [*Klebsiella variicola* At-22]	D	133	2.44	gi|288,933,584	35.5	5.20	73	99.523		
161	Hypothetical protein MGG_02748 [*Magnaporthe oryzae* 70–15]	D	54	2.26	gi|145,610,056	160.7	6.32	67	98.103	19	
162	Tubulin alpha-B chain [*Neurospora crassa*]	D	52	2.15	gi|46,397,830	49.9	5.05	72	99.477	49	99.572
163	Hypothetical protein MGG_00871 [*Magnaporthe oryzae* 70–15]	D	77	2.11	gi|39,973,965	54.4	6.40	69	98.775		
164	Citrate synthase [*Neurospora crassa*]	D	72	−2.15	gi|30,316,357	52.0	8.10	314	100	224	100
165	Vacuolar ATP synthase subunit B [*Magnaporthe oryzae*70–15]	D	53	−2.21	gi|39,942,328	56.7	5.33	95	99.997	18	
166	Fructose-1,6-bisphosphatase [*Aspergillus clavatus* NRRL 1]	D	134	−2.24	gi|119,400,142	38.8	5.30	97	99.998	83	100
167	V-type proton ATPase catalytic subunit [*Neurospora crassa*]	D	130	−2.26	gi|137,461	67.1	5.32	65	97.381	50	99.561
168	Hypothetical protein [*Podospora anserina* S mat+]	D	101	−2.42	gi|171,687,995	88.8	6.57	86	99.977		
169	Beta glucosidase, putative [*Aspergillus clavatus* NRRL 1]	D	47	−2.53	gi|119,396,244	84.3	5.60	61	93.571	47	99.405
170	Hypothetical protein CHGG_05845 [*Chaetomium globosum* CBS 148.51]	D	82	−2.54	gi|116,192,255	43.4	5.78	88	98.698		
171	Predicted protein [*Nematostella vectensis*]	D	118	−2.62	gi|156,376,551	41.4	6.27	83	95.787		
172	Heat shock protein 60, mitochondrial precursor [*Magnaporthe oryzae* 70-15]	D	110	−2.77	gi|145,608,376	61.8	5.83	125	100	77	100
173	Probable succinyl-CoA ligase [*Neurospora crassa*]	D	71	−3.49	gi|74,665,374	34.7	9.10	88	99.987	55	99.902
174	Heat shock protein 70 (hsp70) [*Aspergillus clavatus* NRRL 1]	D	99	−3.59	gi|119,403,457	72.5	5.81	82	99.941		
175	Adenosylhomocysteinase [*Magnaporthe oryzae* 70-15]	D	78	−3.65	gi|39,940,170	48.9	5.94	80	99.907	35	90.312
176	Myosin, heavy polypeptide 13, skeletal muscle [*Xenopus (Silurana) tropicalis*]	D	96	−4.07	gi|55,742,222	222.7	5.54	95	99.758		
177	Hypothetical protein [*Podospora anserina* S mat+]	D	94	−4.31	gi|189,091,826	63.7	5.94	64	96.126	57	99.950
178	Isoleucyl-tRNA synthetase [*Klebsiella variicola* At-22]	D	48	−4.39	gi|288,937,224	104.4	5.64	67	98.347		
179	Hypothetical protein [*Podospora anserina* S mat+]	D	49	−5.61	gi|171,688,418	47.6	8.94	69	98.883		
180	Hypothetical protein [*Podospora anserina* S mat+]	D	2	−9.20	gi|171,690,254	117.5	6.22	68	98.625		
181	Glyceraldehyde-3-phosphate dehydrogenase [*Beauveria bassiana*]	E/E	42/55	5.14/-2.57	gi|50,659,022	36.1	6.54	174/189	100/100	65/31	99.553/0
182	212L [Invertebrate iridescent virus 6]	E	3	41.79	gi|15,078,924	43.0	5.88	63	99.605		
183	Unnamed protein product [*Kluyveromyces lactis*]	E	6	24.36	gi|50,303,991	33.2	5.24	90	99.179	39	0
184	Translation elongation factor EF-Tu, putative [*Aspergillus clavatus* NRRL 1]	E	10	15.62	gi|119,397,185	48.3	6.52	44	0	36	95.982
185	Heat shock protein 70 kDa [*Hypocrea lixii*]	E	14	12.89	gi|167,843,281	71.0	5.05	352	100	197	100
186	Copper-zinc superoxide dismutase [*Cordyceps militaris*]	E	22	8.16	gi|26,000,295	15.7	6.28	77	99.810	53	99.819
187	Cytochrome P450 [*Aspergillus clavatus* NRRL 1]	E	28	7.10	gi|119,396,129	60.0	9.00	64	96.626		
188	Protein disulphide isomerase [*Hypocrea jecorina*]	E	51	4.17	gi|3,288,650	54.6	4.83	82	94.697	60	97.205
189	Pc21g12150 [*Penicillium chrysogenum* Wisconsin 54–1255]	E	60	3.07	gi|255,954,987	89.7	6.36	68	98.687		
190	SPEE_NEUCR RecName: Full = Spermidine synthase	E	67	2.37	gi|8,134,725	33.1	5.54	72	99.414	18	0
191	Hypothetical protein [*Podospora anserina* S mat+]	E	44	2.05	gi|171,695,866	21.9	5.39	61	92.950	49	99.741
192	Chain R, Isometrically Contracting Insect Asynchronous Flight Muscle	E	74	−2.30	gi|295,789,252	41.4	5.16	68	99.875	13	0
193	Vacuolar ATP synthase catalytic subunit A, putative [*Talaromyces stipitatus* ATCC 10,500]	E	82	−2.37	gi|242,791,712	76.8	5.53	88	98.637	37	0
194	Malate dehydrogenase, mitochondrial precursor [*Chaetomium globosum* CBS 148.51]	E	80	−2.59	gi|116,197,148	35.3	8.63	194	100	57	97.025
195	Pol polyprotein [Human immunodeficiency virus type 1]	E	87	−2.99	gi|13,738,350	45.5	8.96	83	96.158		
196	Unnamed protein product [*Podospora anserina*]	E	90	−3.41	gi|171,696,284	59.3	9.24	118	99.999	18	0
197	Cell division control protein 10 [*Neurospora crassa* OR74A]	E	91	−6.35	gi|85,076,041	38.6	7.21	111	99.994	61	96.391
198	Chaperone protein DnaK, putative [*Stigmatella aurantiaca* DW4/3–1]	E	54	−9.77	gi|115,379,880	45.6	9.34	87	98.539		

A-E, the same as in Figure 2.

Though *Hirsutella sinensis* is usually considered as the anamorph of *C. sinensis* (Chen et al. 2004; Li et al. 2006a; Zhong et al. 2010), cultivation of its fruiting body is still very difficult. Alternatively, proteomic comparison of the fruiting body from natural *C. sinensis* and mycelia of *H. sinensis* was determined to explore the potential proteins related to the formation of the fruiting body. There were 1983 protein spots detected in both the fruiting body of natural *C. sinensis* and mycelia of *H. sinensis*, which was also much more than previous reports, 188 spots in mycelia of *H. sinensis* (Jin 2005) and 630 spots in mycelia of an isolated fungal strain of *C. sinensis* (Kao 2006). Among the detected spots, 559 (28.2%) and 401 (20.2%) spots, respectively, were up-regulated and down-regulated in fruiting bodies of natural *C. sinensis* (Threshold mode: 2.0-fold) (Figure 2), and 64 proteins in 69 protein spots (35.9%) out of 192 picked spots (Figure 3) were successfully identified (Table 1). There was no spot was identified in previous report (Kao 2006).

#### Differential proteins in late/early stages of the fruiting body and fruiting body/mycelia of *C. militaris*

*C. militaris* is a major species of *Cordyceps* widely used in the market. The fruiting body of *Paecilomyces militaris*, anamorph of *C. militaris*, is easily formed in cultured media. It is great help to know the proteins expression during fruiting body formation based on the investigation of proteins in mycelia, early and late stages of the fruiting body of *C. militaris*. As a results, 2175 protein spots were detected in both early and late stage fruiting bodies of *C. militaris*, 295 (13.6%) and 234 (10.8%) out of the detected spots were up-regulated and down-regulated in the late stage fruiting body, respectively (Threshold mode: 2.0-fold) (Figure 2). Among 95 picked spots (Figure 3), 40 proteins in 48 protein spots (50.5%) were successfully identified (Table 1). On the other hand, there were 2227 protein spots detected in both the fruiting body and mycelia of *P. militaris*, and 523 (23.5%) and 449 (20.2%) of detected spots were up-regulated and down-regulated, respectively, in the fruiting body of *C. militaris* (Threshold mode: 2.0-fold) (Figure 2). Finally, 33 proteins in 40 protein spots (44.4%) out of 90 picked spots (Figure 3) were successfully identified, which included 18 up-regulated and 22 down-regulated proteins in the late stage fruiting body of *C. militaris* (Table 1).

#### *Differential proteins between the fruiting body and mycelia of* C. memorabilis

*C. memorabilis* is one of the species of *Cordyceps* genus. The fungus, *Isaria farinose*, anamorph of *C. memorabilis*, could form the fruiting body under laboratory conditions. Therefore, comparison of proteins in the fruiting body and mycelia of *I. farinose* is also helpful to well understand the molecular mechanism of formation of natural *C. sinensis*. By DIGE analysis, 2275 protein spots were detected in both the fruiting body and mycelia of *I. farinosa*. Among the detected spots, 554 (23.9%) and 430 (18.9%) spots were, respectively, up-regulated and down-regulated in the fruiting body (Threshold mode: 2.0-fold) (Figure 2), and 23 proteins in 25 protein spots (29.8%) out of 84 picked spots (Figure 3) were successfully identified (Table 1).

Totally, 115 differential expression proteins in 134 protein spots were found in both fruiting bodies and mycelia of three species of *Cordyceps* (*C. sinensis, C. memorabilis*, and *C. militaris*). It was worth to note that enolase/putative enolase up-regulated, while ATP synthase down-regulated coincidentally in all fruiting bodies of *Cordyceps*.

For natural *C. sinensis*, acetaldehyde dehydrogenase, beta-tubulin, elongation factor 2, enolase, malate dehydrogenase, heat shock 70 kDa protein and hypothetical protein FG09893.1 were simultaneously up-regulated in both fruiting bodies of *C. sinensis* versus sclerotia and fruiting bodies of *C. sinensis* versus mycelia of *H. sinensis*. These results suggested that the seven proteins played important roles during the fruiting body formation of natural *C. sinensis*. Besides, the enolase or putative enolase was up-regulated, while ATP synthase was down-regulated coincidentally in fruiting bodies of *C. memorabilis* and *C. militaris* than that in corresponding mycelia of *I. farinosa* and *P. militaris*, which showed that the enolase and ATP synthase were the most important proteins for fruiting body formation of *Cordyceps*. The beta-tubulin was also up-regulated in the fruiting bodies of *C. militaris* as well as in natural *C. sinensis*. Although malate dehydrogenase and acetaldehyde dehydrogenase were up-regulated in fruiting bodies of natural *C. sinensis*, they were down-regulated in the fruiting bodies of *C. memorabilis.*

Apart from the shared proteins, most of the differential expression proteins were non-shared proteins in the fruiting bodies of *C. sinensis* versus sclerotia, and fruiting bodies of *C. sinensis* versus mycelia of *H. sinensis*. The reasons may arise from different samples (natural sclerotia and cultured mycelia) and a small probability of picking the same spot in different gels under blind screening (the picked spot must possess simultaneously higher differential expression and intact three-dimensional separation map).

### Biological activities of proteins during fruiting body formation of C. sinensis

Fruiting body formation of filamentous fungi is one of the most complex developmental processes. It not only requires the aggregation of hyphae to form three-dimensional structures, and leads to the differentiation of a number of fruiting bodies-specific cell types not present in the vegetative mycelium (Nowrousian et al. 2007), but also requires precise integration of a number of fundamental biological processes under special environmental conditions and is controlled by many developmentally regulated genes (Pöggeler et al. 2006).

#### Camp signal pathway in fruiting body formation of C. sinensis

Two cytoplasmic signalling branches, the cAMP-dependent protein kinase (PKA) and mitogen-activated protein kinase (MAPK) pathway, regulate gene expression that finally leads to fruiting body formation. Indeed, MAPK genes are required for fruiting in *Aspergillus nidulans, Neurospora crassa*, and *Lentinula edodes*, where MAPK kinase (Demeke et al. 1997), MEK kinase (MEKK) and MAPK involve in the pathway (Szeto et al. 2007). However, no proteins related to the MAPK pathway were identified in this study. Similarly, orthologous MAPK genes were not transcribed (CCM_04200 vs. AN1017) or transcribed at low levels (CCM_01235 vs. NCU02393) by *C. militaris* (Zheng et al. 2011). The reasons may include: 1) Different higher fungi might depend on different signal pathways in fruiting body development; 2) Although the related MAPK proteins involved in fruiting body development of *C. sinensis*, they were not successfully identified for the limited *Cordyces* database.

In the two signalling cascades, either heterotrimeric G proteins or ras and ras-like proteins relay extracellular ligand-stimulated signals to the cytoplasm (Pöggeler et al. 2006). Indeed, two main upstream signalling regulators of adenylyl cyclase, guanine nucleotide-binding protein (G proteins) beta subunit-like protein and GTP-binding protein Ran, increased in the fruiting body of *C. sinensis* (Table 2). G proteins can interact with adenylyl cyclases and catalyse the formation of cAMP (D’Souza and Heitman, 2001; Kamerewerd et al. 2008). GTP-binding protein Ran belongs to the superfamily of Ras proteins and is crucial regulator of adenylyl cyclase (Schlenstedt et al. 1997; Seewald et al. 2003). Rab GDP-dissociation inhibitor (RabGDI) is a key regulator of Rab/Ypt GTPases that controls the distribution of active GTP and inactive GDP-bound forms between membranes and cytosol (Rak et al. 2003). Actually, RabGDI was up-regulated in the fruiting body of *C. sinensis* (Table 2). The same result has also been observed in the fruiting body of mushroom *L. edodes* (Sakamoto et al. 2009). In addition, GAF domain protein with 3ʹ, 5ʹ-cyclic-AMP phosphodiesterase activity, downstream signalling regulator of adenylyl cyclase, catalyses cAMP to AMP (De Oliveira et al. 2007) decreased in the fruiting body of *C. sinensis*. Finally, increased biosynthesis and decreased degradation of cAMP result in accumulation of cAMP in the fruiting body of *C. sinensis*. As a signalling factor, cAMP plays an important role in controlling fruit body formation (Kinoshita et al. 2002; Palmer and Horton 2006). It is closely related to the onset of fruiting body development in *L. edodes* (Miyazaki et al. 2005). The level of cAMP in dikaryotic mycelia of *Schizophyllum commune* reached peak before primodium formation, and then gradually increased until the final stage of fruit body formation (Kinoshita et al. 2002). Light causes an increase of cAMP level in fungi *Coprinus macrorhizus* and *S. commune*, and induces their fruiting body formation (Kinoshita et al. 2002). It has been confirmed that *C. militaris* fail to form the fruiting body without light. Moreover, cAMP also regulates the expression of a large number of genes required for fruiting body formation of *Dictyostelium discoideum* (Bishop et al. 2002). Therefore, the cAMP signal pathway should involve in fruiting body development of *C. sinensis*.

**Table 2.** Identified proteins related to fruiting of *C. sinensis* and other microorganisms.

No.	Protein Name	Group	Role in fruiting body development	Microorganism	References
1	Enolase or putative enolase	A↑, B↑, C↑, E↑, D↑↓	Enolase, also known as phosphopyruvate hydratase, is a metalloenzyme responsible for the catalysis of the conversion of 2-phosphoglycerate to phosphoenolpyruvate, might involve the fruiting body formation of *Cordyceps sinensis.*	*Cordyceps sinensis, C. militaris*, and *C. memorabilis*	
2	Heat shock proteins 70 (hsp70)	A↑, B↑, C↑↓, D↓, E↑	Sudden change of temperature (heat shock or cold shock) or other adverse environmental conditions can stimulate living organisms to produce heat shock proteins (Hsps) for protection and cell repairmen activities. Heat shock induces some proteins expression and perhaps involve in fruiting body formation and sporulation of *Myxococcus xanthus.*	*Myxococcus xanthus*	(Chaffin et al. 1998; Otani et al. 2001)
3	Putative hsp70 chaperones	A↑, B↓, C↓, D↓			
4	Heat shock 70 kd protein cognate 1	A↓, C↑			
5	Heat shock proteins 90	A↑↓, C↑, D↓	A gene encoding Hsp90 homolog involves in both sexual development and vegetative growth of *Podospora anserina*		(Loubradou et al. 1997)
6	Heat shock protein 60	C↓, D↓			
7	Acetaldehyde dehydrogenases	A↑, B↑, C↑↓, E↓	Acetaldehyde dehydrogenase is induced by heat shock in *Myxococcus xanthus*, and is related to fruiting body formation of the mushroom *Flammulina velutipes.*	*Myxococcus xanthus, Flammulina velutipes*	(Otani et al. 2001; Yoon et al. 2002)
8	Malate dehydrogenases	A↑, B↑, C↓, E↓	Malate dehydrogenase is related to the sporulating during fruiting body development in *Pleurotus ostreatus.*	*Pleurotus ostreatus*	(Chakraborty et al. 2003)
9	Tubulins	A↑, B↑, C↓, D↑	Tubulins T1 and T2 are strongly increased during fruiting body formation of fungus *Physarum polycephalum.*	*Physarum polycephalum*	(Putzer et al. 1984; Poetsch et al. 1989)
10	Actins	A↑↓, B↑, C↑↓	An actin is decreased during fruiting body formation of fungus *Physarum polycephalum.*	*Physarum polycephalum*	(Putzer et al. 1984; Poetsch et al. 1989)
11	Predicted similar to actin-5C isoform 1, beta actins, actin 6	A↓			
12	ATP synthases	A↑, B↓, C↑, D↓, E↓	ATP synthase is induced by heat shock in *Myxococcus xanthus*, and is induced during fruit body development and maturation of *Agaricus bisporus.*	*Agaricus bisporus*	(De Groot et al. 1997; Otani et al. 2001)
13	Elongation factors 2	A↑, B↑	Transcript of elongation factor 2 highly expresses in the fruiting body cDNA library of medicinal fungus *Ganoderma lucidum*; the elongation factor 1A controls the fruiting body formation of *Podospora anserina*, interacts with actin and tubulin, activates degradation of some proteins, and is probably involved in signal transduction and cell cycle regulation; the gene of elongation factor 1 is one of developmentally specific genes in the primordium of *Lentinula edodes.*	*Ganoderma lucidum, Podospora anserina, Lentinula edodes*	(Silar et al. 2001; Miyazaki et al. 2005; Luo et al. 2010)
14	Elongation factor 3	A↑			
15	Putative translation elongation factor EF-Tu	E↑			
16	Mannose-1-phosphate guanyltransferase	A↑, B↓	The overexpressed mannose-1-phosphate guanyltransferase promotes increase of GDP-mannose in fungus *Trichoderma reesei*, and GDP-mannose might play a major regulatory role in protein glycosylation.	*Trichoderma reesei*	(Zakrzewska et al. 2003)
17	Cobalamin-independent methionine synthase	A↑, C↑↓	The cobalamin-independent methionine synthase only be oberserved in conidia rather than in the mycelium of entomopathogenic fungus *Metarhizium acridum.*	*Metarhizium acridum*	(Barros et al. 2010)
18	Septin	A↑, C↑	The septin is strongly induced during fruit body development and maturation of *Agaricus bisporus.*	*Agaricus bisporus*	(De Groot et al. 1997)
19	Spermidine synthase	C↑, E↑	Development of spermidine synthase (*spsA*) null cells grown in the absence of spermidine produced fruiting bodies of *Dictyostelium discoideum* that have abnormally short stalks.	*Dictyostelium discoideum*	(Guo et al. 1999)
20	Guanine nucleotide-binding protein (G protein) subunit beta-like protein	B↑, C↑↓	G proteins are essential for growth, asexual and sexual development, and virulence in both animal and plant pathogenic filamentous species. In fungi, G proteins play integral roles for cell growth/division, mating, cell-cell fusion, morphogenesis, chemotaxis, virulence establishment, pathogenic development and secondary metabolite production.	*Aspergillus nidulans*	(Yu 2006; Li et al. 2007)
21	GTP-binding nuclear protein Ran, putative	B↑	GTP-binding protein Ran belongs to the superfamily of Ras proteins and is crucial regulator of adenylyl cyclase.	*Saccharomyces cerevisiae*	(Schlenstedt et al. 1997; Seewald et al. 2003)
22	Glyceraldehyde-3-phosphate dehydrogenase	D↓, E↑↓	The glyceraldehyde-3-phosphate dehydrogenase gene *GAPDH* was expressed in both mycelia and fruiting bodies, suggesting that the *GAPDH* gene product is a heat shock protein which might be involved in the developmental phase of the *Lentinus polychrous.*	*Lentinus polychrous*	(Thanonkeo et al. 2010)
23	Rab GDP-dissociation inhibitor	A↑, C↓	Rab GDP-dissociation inhibitor, a key regulator of Rab/Ypt GTPases that controls the distribution of the active GTP and inactive GDP-bound forms between membranes and cytosol, is up-regulated in fruiting body of mushroom *Lentinula edodes.*	*Lentinula edodes*	(Rak et al. 2003; Sakamoto et al. 2009)
24	Serine proteases	A↓, B↑	The serine proteases play an important roles in the pathogenic fungus during the penetration and colonisation of their hosts.	*Cordyceps sinensis*	(Li et al. 2006b; Zhang et al. 2008)
25	Formate dehydrogenase	B↓, E↑			
26	UTP-glucose-1-phosphate uridylyltransferase	A↑	The UTP-glucose-1-phosphate uridylyltransferase is a developmentally regulated enzyme which involves in trehalose, cellulose, and glycogen synthesis in fungus *Dictyostelium discoideum.*	*Dictyostelium discoideum*	(Fishel et al. 1982; Bishop et al. 2002)
27	Mannitol-1-phosphate 5-dehydrogenase	B↑	The mannitol-1-phosphate 5-dehydrogenase is proposed as the major enzyme for mannitol biosynthesis, and the increase of mannitol is related to the fruiting body initiation and development of *Agaricus bisporus.*	*Agaricus bisporus*	(Kulkarni, 1990; Vélëz et al. 2007)
28	Chorismate mutase	B↓	The mutant strains of *Aspergillus nidulans* which have been knocked out the chorismate mutase gene *aroC*, decreases the capacity for fruit body formation and ascosporogenesis.	*Aspergillus nidulans*	(Krappmann, and Braus, 2003)
29	*O*-acetylhomoserine sulfhydrylase (homocysteine synthase)	A↑	The homocysteine synthase plays an important role in the cysteine synthesis in *Tuber borchii* and may involve in the formation of fruiting body.	*Tuber borchii*	(Zeppa et al. 2010)
30	UDP-N-acetylglucosamine pyrophosphorylase	A↑	The UDP-*N*-acetylglucosamine pyrophosphorylase is a major regulatory enzyme in amino sugar synthesis during cyst wall (encystment) formation of *Giardia.*	*Giardia*	(Bulik et al. 2000)
31	Mago nashi protein	B↑	The mago nashi protein participates fungi development and abundantly expresses in natural fruiting bodies of medicinal fungus *Antrodia cinnamomea.*	*Antrodia cinnamomea*	(Chu et al. 2009)
32	T-complex protein 1 subunit zeta	A↑	The T-complex protein is the developmentally specific gene product in mature fruiting body of *Lentinula edodes.*	*Lentinula edodes*	(Miyazaki et al. 2005)
33	Inorganic pyrophosphatase or putative inorganic diphosphatase	A↑, C↑			
34	6-phosphogluconate dehydrogenase	C↑, D↑↓			
35	Hypothetical protein (gi|171,690,628)	B↓, C↓, D↓	Although their bioactivities of these hypothetical proteins are unknown, they might play important roles in the fruiting body development of *C. sinensis.*		
36	Hypothetical protein (gi|171,683,195)	B↓, C↓			
37	Hypothetical protein FG09893.1	A↑, B↑			

↑ represents up-regulated, ↓ represents down-regulated, ↑↓ represents up-regulated and down-regulated.

## Heat shock proteins responded to environmental stress

In fungal kingdom, fruit body formation usually could not happen until some severe stressors occur. In nature, these stressors are heat and cold, fire and flood, or nutrient deficiency (Holliday and Cleaver 2008). A sudden change in temperature (heat shock or cold shock) or other adverse environmental conditions can stimulate living organisms to produce heat shock proteins (Hsps) for protection and cell repairmen activities. Some Hsps play important roles in all major growth-related processes including cell division, DNA synthesis, transcription, translation, protein folding and transportation, and membrane translocation (Chaffin et al. 1998). Generally, heat shock proteins Hsp70, Hsp70 chaperone and Hsp 90 in the fruiting body of *C. sinensis* had higher expression, and a similar change was also found in the mature fruiting body of *C. militaris* (Table 2). Except *Cordyceps*, in *Podospora anserina*, a gene encoding Hsp90 homolog involves in both sexual development and vegetative growth (Loubradou et al. 1997). Under certain environmental stresses, dikaryotic mycelia aggregate to form primordium, which marks the beginning of fruit body development (Chum, et al. 2008). Heat shock treatment accelerates the fruiting body formation and sporulation of *Myxococcus xanthus* because heat shock induces some proteins expression and perhaps involve in fruiting body formation and sporulation (Otani et al. 2001), which well explained why some fungal cultures cannot produce fruit bodies without temperature downshift or light illumination (Yoon et al. 2002). As far as we know, natural *C. sinensis* grows in Qinghai-Tibetan Plateau, where the temperature difference between day and night can reach about 20ºC during fruiting body formation and development season. Therefore, it is reasonable to speculate that heat shock proteins (Hsps) highly express during fruiting body formation and development. On the other hand, Hsps are also immunodominant antigens and major targets of host immune response during different types of infection (Chaffin et al. 1998), which is helpful to better understand why some Hsps show higher expression in sclerotium. It could be presumed that the host larva produces Hsps when it is infected by hyphal or spore of fungus.

## Proteins involved in carbohydrate metabolism

Carbohydrate catabolism not only provides energy for hyphal growth but also supplies carbon skeleton to other metabolisms (Deveau et al. 2008), which is significantly changed during fruiting body initiation and development of primordia into the mature fruiting body (Kulkarni, 1990).

### Proteins involved in the glycolytic pathway and tricarboxylic acid cycle (TCA)

The fructose-bisphosphate aldolase, enolase and pyruvate kinase of the glycolytic pathway, as well as malate dehydrogenase of tricarboxylic acid cycle (TCA), were shown higher expression in the fruiting body of *C. sinensis* (Table 1). It was very intriguing that putative enolase was also up-regulated in fruiting bodies of *C. memorabilis* and *C. militaris*, and enhanced in the mature fruiting body of *C. militaris* (Table 2). These results suggest that enolase may play an important role during fruiting body formation and development of *Cordyceps*. It is consistent with that glycolysis and TCA cycles are the major pathways of glycometabolism in sporulating stage of fruiting body development in *Pleurotus ostreatus* (Chakraborty et al. 2003). In contrast to *C. sinensis*, malate dehydrogenase showed lower expression in the fruiting body of *C. memorabilis* and mature one of *C. militaris*, which may attribute to the different formation mechanisms of individual fungus because the fruiting body of both *C. memorabilis* and *C. militaris* could be produced under the same culture conditions, but *C. sinensis* failed to develop its fruiting body.

### Proteins involved in the glyoxylate pathway

Pyruvate kinase (PK) and aldehyde dehydrogenase are putative indole receptor proteins involved in multicellular development which are essential for fruiting body formation in *Stigmatella aurantiaca* (Stamm et al. 2005). Acetaldehyde dehydrogenase of the glyoxylate pathway, which can be induced by heat shock in *M. Xanthus* (Otani et al. 2001), is related to fruiting body formation of mushroom *Flammulina velutipes* (Yoon et al. 2002). These enzymes increased in the fruiting body of *C. sinensis* and the mature fruiting body of *C. militaris* (Table 2) suggested these enzymes might involve in fruiting body development.

### Proteins involved in the mannitol pathway

Mannitol-1-phosphate 5-dehydrogenase is proposed as main enzyme for mannitol biosynthesis (Vélëz et al. 2007), and the enzyme abundance in the fruiting body of *C. sinensis* was near 13-fold higher than that in mycelia (Table 1). As a result, it may increase the content of mannitol in natural *C. sinensis* (Wang et al. 2009; Guan et al. 2010). Increased mannitol is related to fruiting body initiation and development of *A. bisporus* (Kulkarni, 1990), and the mannitol content in the fruiting body of *A. bisporus* is about 8–20 times higher than that in mycelia (Hammond and Nichols 1976; Wannet et al. 2000).

### Proteins involved in the trehalose pathway

The trehalose pathway is clearly shown by enhanced expression of UTP-glucose-1-phosphate uridylyltransferase (Uridine diphosphoglucose pyrophosphorylase) in the fruiting body, which is a developmental regulation enzyme involving in trehalose, cellulose and glycogen synthesis in fungus *D. discoideum* (Fishel et al. 1982; Bishop et al. 2002). It is essential for fungus to complete its life cycle, and it increases 3-fold at the stage of fruiting body formation than that in vegetative growth and early stage of differentiation (Fishel et al. 1982). The UTP-glucose-1-phosphate uridylyltransferase in the fruiting body of *C. sinensis* was about 9-fold higher than that in sclerotium (Table 1), which may contribute to the higher trehalose content in the fruiting body of *C. sinensis* (Wang et al. 2009).

Immunoglobulin heavy chain-binding protein homolog was overexpressed in the fruiting body of *C. sinensis* rather than in mycelium (Table 1). Homolog gene of immunoglobulin heavy chain-binding protein (78 kDa glucose-regulated protein) is differentially expressed in primordium of mushroom *L. edodes*, which can be inferred that glucose-regulated protein involved in fruit body development under certain environmental stresses (Chum, et al. 2008).

### Proteins involved in the mannose pathway

The content of mannose-1-phosphate guanyltransferase in the fruiting body of *C. sinensis* was higher than that in sclerotium, but lower than that in mycelium (Table 1). The overexpressed mannose-1-phosphate guanyltransferase promotes increase of GDP-mannose in fungus *Trichoderma reesei*. GDP-mannose was effectively utilised by mannnosyltransferases and resulted in hypermannosylation of secreted proteins in both N and O glycosylation, which indicated that GDP-mannose might play a major regulatory role in protein glycosylation in *T. reesei* (Zakrzewska et al. 2003).

## Proteins involved in energy metabolism

Fruiting body developmental programme needs more energy than simple vegetative growth (Busch and Braus 2007). ATP synthase, which can be induced by heat shock (Otani et al. 2001), is high expressed in fruiting body development and maturation of *A. bisporus* (De Groot et al. 1997). Obviously, it is noticed that vacuolar ATP synthase catalytic subunit A and inorganic pyrophosphorylase were higher in the fruiting body than those in sclerotium of *C. sinensis*. Especially, inorganic pyrophosphorylase, which can catalyse degradation of pyrophosphate and release energy, in the fruiting body of *C. sinensis* was about 6-fold higher than that in sclerotium (Table 1).

## Proteins involved in protein synthesis and degradation

Elongation factors, eEF1A, eEF2 and eEF3, serve an essential function in translation cycle of protein synthesis in fungi. The transcript of eEF2 is also highly expressed in fruiting body cDNA library of medicinal fungus *Ganoderma lucidum* (Luo et al. 2010). In addition, the gene of elongation factor 1 is one of developmentally specific genes in primordium of *L. edodes* (Miyazaki et al. 2005), and eEF1A controls fruiting body formation of *P. anserina*, interacts with actin and tubulin to activate some proteins degradation and is probably involved in signal transduction and cell cycle regulation (Silar et al. 2001). Some factors of protein synthesis, including eukaryotic initiation factor 4A (eIF4A), elongation factors eEF2 and eEF3, and ribosomal L18ae protein family, were expressed at higher levels in the fruiting body of *C. sinensis* than that in sclerotium and mycelium. Similarly, the abundance of translation elongation factor EF-Tu in the fruiting body of *C. memorabilis* was higher than that in mycelium (Tables 1 and 2). Protein synthesis activity is very active during fruiting body formation of *C. sinensis*, which is consistent with a higher protein level (30.4%) in the fruiting body of natural *C. sinensis* than that (14.8%) in fermented mycelium (Hsu et al. 2002).

Besides proteases play an important role in turnover of nitrogenous compounds (e.g. protein and amino acids) during fruiting body formation (Terashita et al. 1998), proteolytic enzymes such as serine proteases, proteasome component PUP3, and probable proteasome subunit alpha were also highly expressed in the fruiting body rather than in mycelium and sclerotium of *C. sinensis* (Table 1). Similarly, although serine protease is active in all stages of fruiting body development in *Coprinopsis cinerea*, its expression is the most abundant during young tissue development (Heneghan et al. 2009). High abundance of serine protease in the fruiting body may decompose useless proteins for fruiting body development. But, serine protease in sclerotium may be beneficial for fungus to infect its host through digesting protein component of insect cuticles (Li et al. 2006b; Zhang et al. 2008). Two cuticle-degrading serine proteases from mycelium of fungus *C. sinensis* strain CS2 has been obtained (Zhang et al. 2008). These results show that the process of protein turnover is more active during fruiting body formation.

Amino acid status also has a strong impact on cleistothecium development in *A. nidulans* (Krappmann, and Braus, 2003). It is reported that the total level of amino acids in the fruiting body (16.4%) is higher than that in fermented mycelia (9.23%) (Hsu et al. 2002). Some enzymes catalysing amino acid synthesis, such as *O*-acetylhomoserine sulfhydrylase, cobalamin-independent methionine synthase and chorismate binding enzyme were highly expressed in the fruiting body of *C. sinensis* (Table 1), which indicated that amino acid biosynthesis is active in fruiting body formation. Indeed, homocysteine synthase (*O*-acetylhomoserine sulfhydrylase), which was over-expressed in mature ascoma of fungus *Tuber borchii*, might be involved in its fruiting body formation (Zeppa et al. 2010). Cobalamin-independent methionine synthase can be only observed in conidia rather than in mycelia of entomopathogenic fungus *Metarhizium acridum* (Barros et al. 2010). The enzyme is inducible by heat and estrogen in fungus *Candida albicans* (Burt et al. 1999). While homologous chorismate binding enzymes can catalyse the initial biosynthesis of tryptophan, menaquinone and siderophores (Zwahlen et al. 2007). Especially, chorismate mutase is the first enzyme of the branch of the shikimate pathway which catalyses a necessary step in biosynthesis of aromatic amino acids. Aromatic amino acids are not only the essential composition of proteins but also crucial precursors for many secondary metabolites (Pudelski et al. 2009). Fruit body formation and ascosporogenesis of *Aspergillus nidulans* significantly decreased with chorismate mutase gene *aroC* knocked out (Krappmann, and Braus, 2003). Unfortunately, chorismate mutase decreased in the fruiting body of *C. sinensis* rather than mycelia of *H. sinensis*. The mechanism need further study.

## Proteins involved in cell reconstruction

It is a very complicated transformation that from wire-like hyphal filaments into complex and sometimes container-like fruit bodies, which is necessary for the transformation to reconstruct major cells (Busch and Braus 2007).

Actins are highly conserved proteins involved in various types of cell motility, and tubulins are involved in complex structures like the mitotic spindle, centrioles, cilia, flagella and cytoskeleton (Poetsch et al. 1989). During fruiting body formation of fungus *Physarum polycephalum*, the most prominent syntheses of an actin decreased while two tubulins T1 and T2 were strongly increased (Putzer et al. 1984; Poetsch et al. 1989). Increased tubulins suggest the cellular reconstruction is active during fruiting body formation. Actually, predicted similar to Actin-5C isoform 1, actin 6, beta actins were down-regulated, but beta tubulins were consonantly up-regulated in the fruiting body of *C. sinensis* (Table 1). Alpha 2 tubulin gene was highly expressed in the immature fruiting body of *L. edodes* (Chum et al. 2011), while α-tubulins are constituents of microtubules responsible for cytoskeleton and further cell shape regulation (Juuti et al. 2005). It was noticed that two different tubulins simultaneously decreased in the mature fruiting body of *C. militaris* rather than in early stage, which suggested that tubulins possibly involved in fruiting body initiation rather than maturation.

UDP-*N*-acetylglucosamine pyrophosphorylase is a major enzyme in amino sugar synthesis during cyst wall formation (encystment) of *Giardia* and that its allosteric anabolic activation may shift the equilibrium of this pathway towards UDP-Gal-NAc synthesis (Bulik et al. 2000). UDP-*N*-acetylglucosamine pyrophosphorylase was increased in the fruiting body of *C. sinensis* (Table 1), which was in accordance with the previous report (Jin 2005).

## Proteins involved in cell division control

Septin, as a cell division control protein, is involved in septa formation during cell division, and the highest expression of septin is found in the transitional zone between cap and stipe of mature mushroom *A. bisporus* (De Groot et al. 1998; Zeppa et al. 2002). The expression of this enzyme is strongly induced during fruiting body development and maturation of mushroom *A. bisporus* (De Groot et al. 1997).

Mago nashi proteins, highly conserved among eukaryotes, not only participate in oogenesis, embryogenesis and germ-line sex determination during animal development, but also play important roles in pollen tube growth, root development and spermatogenesis during plant development (Chen and Chu 2010; Lewandowski et al. 2010). They also participate in fungi development and are abundantly expressed in natural basidiomes (fruiting bodies) of medicinal fungus *Antrodia cinnamomea* (Chu et al. 2009).

In this study, septin, mago nashi protein and cell division control protein 3 distinctly increased in the fruiting body of *C. sinensis*, besides higher septin was in the mature fruiting body of *C. militaris* (Table 1). These indicate that cell division contributes to the fruiting body formation.

**Figure 4.**
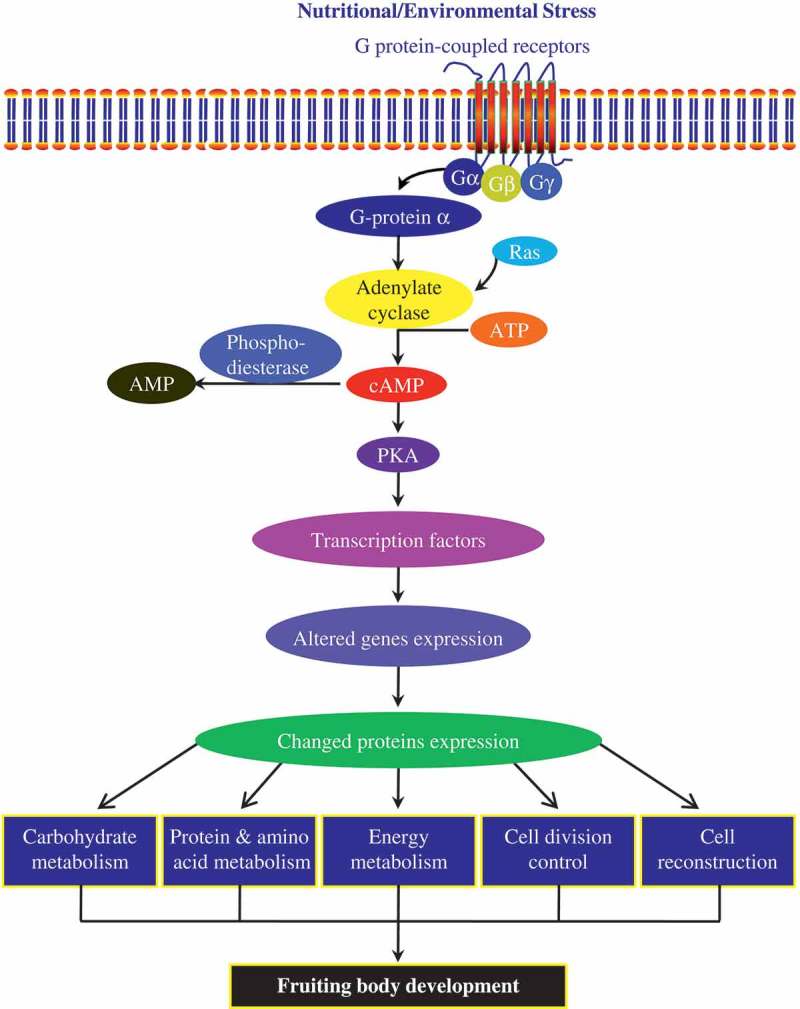
Hypothesised cAMP signal pathways involved in fruiting body formation of *C. sinensis.*

**Figure 5.**
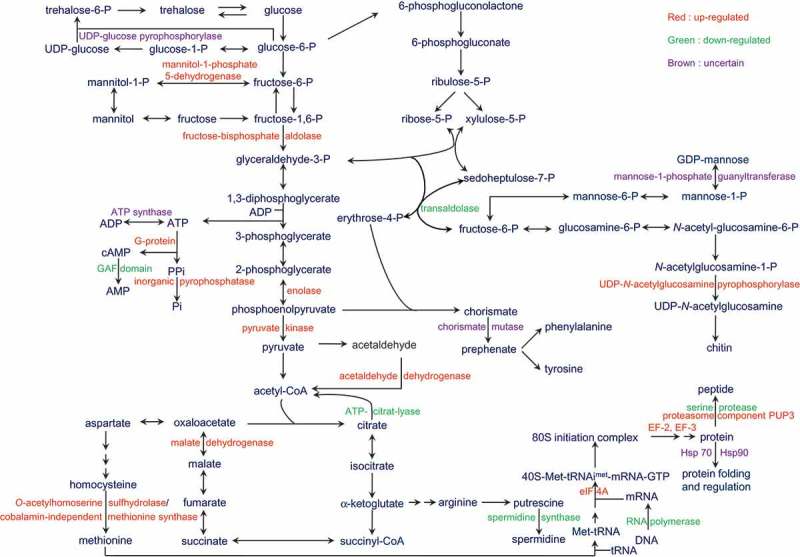
Hypothesised metabolic pathways during fruiting body formation of *C. sinensis.* Up-regulated proteins shown in red, down-regulated proteins shown in green, and uncertained regulated proteins shown in brown.

In summary, this study identified 198 differential expression proteins that may relate to fruiting body development of *Cordyceps*, and 24 proteins have been proven their roles in fruiting body development in other fungi (Table 2). Among the identified proteins, acetaldehyde dehydrogenase, beta-tubulin, elongation factor 2, enolase, malate dehydrogenase, heat shock 70 kDa protein are the key proteins for fruiting body formation and development of *C. sinensis*. Especially, enolase and malate dehydrogenase were first proposed in fruiting body development of mushroom. Besides, the cAMP signal pathway as well as glycometabolism, protein metabolism, energy metabolism, cell division and cell reconstruction are presumed to be related to fruiting body development of *C. sinensis* (Figure 4). A map of metabolic pathways involved in fruiting body development of *C. sinensis* was also hypothesised (Figure 5). It has become evident that fruiting body formation of *C. sinensis* is a highly complex differentiation process and requires precise integration of a number of fundamental biological processes. Although the fruiting body formation mechanisms for all these activities remain to be further elucidated, the study presented here provides a framework for understanding them.
